# Fetal and Maternal Innate Immunity Receptors Have Opposing Effects on the Severity of Experimental Malaria in Pregnancy: Beneficial Roles for Fetus-Derived Toll-Like Receptor 4 and Type I Interferon Receptor 1

**DOI:** 10.1128/IAI.00708-17

**Published:** 2018-04-23

**Authors:** Lurdes Rodrigues-Duarte, Yash Pandya, Rita Neres, Carlos Penha-Gonçalves

**Affiliations:** a Instituto Gulbenkian de Ciência, Oeiras, Portugal; University of South Florida

**Keywords:** TLR4, IFNAR1, pregnancy malaria, fetal innate immunity

## Abstract

Malaria in pregnancy (MiP) is a distinctive clinical form of Plasmodium infection and is a cause of placental insufficiency leading to poor pregnancy outcomes. Maternal innate immunity responses play a decisive role in the development of placental inflammation, but the action of fetus-derived factors in MiP outcomes has been overlooked. We investigated the role of the *Tlr4* and *Ifnar1* genes, taking advantage of heterogenic mating strategies to dissect the effects mediated by maternally and fetally derived Toll-like receptor 4 (TLR4) or type I interferon receptor 1 (IFNAR1). Using a mouse infection system displaying severe MiP outcomes, we found that the expressions of TLR4 and IFNAR1 in the maternal compartment take part in deleterious MiP outcomes, but their fetal counterparts patently counteract these effects. We uncovered that fetal TLR4 contributes to the *in vitro* uptake of infected erythrocytes by trophoblasts and to the innate immune response in the placenta, offering robust protection of fetus viability, but had no sensible impact on the placental parasite burden. In contrast, we observed that the expression of IFNAR1 in the fetal compartment was associated with a reduced placental parasite burden but had little beneficial effect on fetus outcomes. Furthermore, the downregulation of *Ifnar1* expression in infected placentas and in trophoblasts exposed to infected erythrocytes indicated that the interferon-IFNAR1 pathway is involved in the trophoblast response to infection. This work unravels that maternal and fetal counterparts of innate immune pathways drive opposing responses in murine placental malaria and implicates the activation of innate receptors in fetal trophoblast cells in the control of placental infection and in the protection of the fetus.

## INTRODUCTION

Malaria in pregnancy (MiP) shows a wide clinical spectrum that includes severe clinical manifestations such as heightened risks for maternal anemia, fetal abortion, premature delivery, and underweight babies (1–5). It is assumed that the underlying placental inflammation is driven by the maternal immune system and is a central pathological feature in determining placental insufficiency and poor pregnancy outcomes. The receptor-mediated adhesion of infected erythrocytes (IEs) to the fetal trophoblast layer (6, 7) correlates with the recruitment of maternal inflammatory cells, monocytes and macrophages, and with the production of proinflammatory mediators. Increased levels of production of gamma interferon (IFN-γ) and tumor necrosis factor alpha (TNF-α) and enhanced levels of monocyte/macrophage-recruiting factors (MiP-1α and MiP-1β) have been detected in infected placentas (8, 9). This strong inflammatory reaction leads to placental tissue disorganization, which is often accompanied by placental dysfunction and impaired fetal growth (8, 10–12). Clearly, maternally derived proinflammatory factors play a decisive role in placental inflammation, but it remains unclear how fetally derived factors intervene in these pathogenic mechanisms. Here, we made use of a murine infection system displaying severe pregnancy phenotypes in conjunction with heterogenic mating strategies to differentiate pathogenic and protective effects of specific inflammatory receptors expressed in the maternal system or in the fetal compartment. We used this experimental setting to analyze inflammatory mediators known to be involved in the host response to malaria parasites, namely, Toll-like receptor 4 (TLR4) and type I interferon receptor 1 (IFNAR1).

Plasmodium glycosylphosphatidylinositol (GPI) (13, 14) has been shown to stimulate macrophages, dendritic cells (DCs), and endothelial cells through the TLR4 surface receptor, resulting in the increased secretion of proinflammatory cytokines, such as TNF-α, interleukin-1 (IL-1), and IL-12 (14–17). Human genetic studies have associated TLR4 polymorphisms with parasitemia levels in patients with mild malaria (18) and with the risks of clinical malaria (19), severe malaria (20), maternal anemia, and low birth weight in term infants (21). Additionally, bacterial infections in mouse models have been shown to cause placental inflammation and poor pregnancy outcomes in a TLR4-dependent manner (22, 23). Increased fetal death rates (23, 24), preterm delivery rates (23), and fetal weight loss (22) were observed in wild-type (WT) but not in *Tlr4^−/−^* primigravid females exposed to infections by different bacterial species. In these females, systemic blockage of TLR4 signaling significantly reduced preterm delivery (24) and fetal death (23, 24), indicating that the triggering of TLR4 by microbial components is a pathogenesis factor in pregnancy disturbances caused by infection. In addition, it has been shown that peripheral blood TLR4 mRNA levels are significantly increased in women with idiopathic preterm labor, and the TLR4 protein is expressed in fetal trophoblasts (25). TLR4 maternal and fetal gene variants were associated with preterm delivery (26), suggesting that the placental expression of TLR4 plays a role in pregnancy outcomes. Furthermore, it was recently proposed that TLR4 plays a role in experimental MiP (27), prompting the dissection of maternal versus fetal TLR4 effects on MiP.

Recent studies have suggested that innate immune responses to malaria through TLRs and other putative sensors lead to a type I interferon (IFN-I) response (28–31). Strong evidence supports a multilayered role of IFN-I during Plasmodium berghei infection due to the differential activation of multiple components of the IFN signaling pathway, such as the IFN regulatory factor (IRF) family and IFN-stimulated genes (29, 32). Fittingly, IFNAR1 gene polymorphisms have been associated with disease severity and progression to cerebral malaria, especially in children (33–35), and *Ifnar1^−/−^* mice showed significant protection against the development of experimental cerebral malaria (ECM) with a reduced accumulation of CD8^+^ T cells (35–37). Furthermore, it was demonstrated that the expression of IFNAR1 in CD8^+^ T cells is needed to trigger ECM development (35). ECM and MiP share pathogenesis features such as IE adhesion/sequestration (38–41), tissue recruitment of proinflammatory cells (12, 35, 42), and tissue damage (43–46). The involvement of IFNAR1 in innate and adaptive responses to malaria parasites warrants the evaluation of the contributions of maternal and fetal IFNAR1 to MiP pathogenesis.

Here, we used an experimental genetic heterogenic system where homozygous primigravid mice carry heterozygous fetuses, aiming to discern whether the expression of TLR4 and IFNAR1 molecules in either maternal or fetal compartments is implicated in outcomes of malaria in pregnancy. Our data reveal maternal-fetal antagonism in that maternal innate immune pathways respond to placental infection by generating a local proinflammatory environment that contributes to clinical outcomes of MiP, while the same pathways act in the fetal placental tissue to reduce the infection load and secure the survival of the fetus.

## RESULTS

### P. berghei NK65 infection in *Tlr4*- and *Ifnar1*-deficient nonpregnant females.

**FIG 1:**
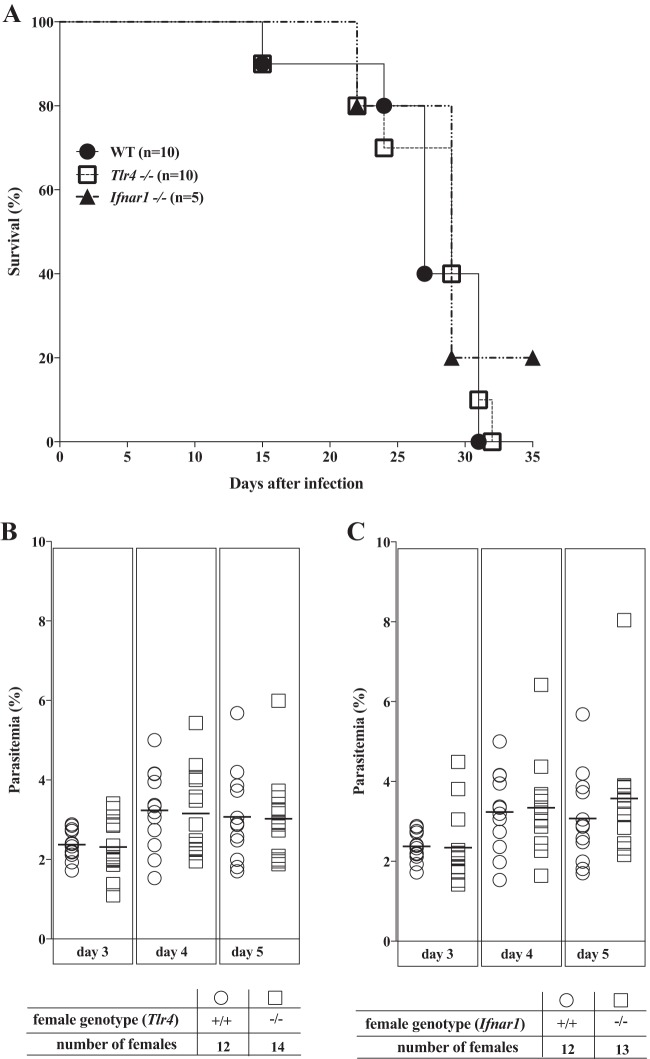
Parasitemia progression and survival in nonpregnant *Tlr4^−/−^* and *Ifnar1^−/−^* females. Adult nonpregnant females were infected i.p. with 10^6^ P. berghei NK65-infected erythrocytes, and peripheral blood parasitemia in the initial phase of infection was determined by FACS analysis using DRAQ5-labeled blood samples at the indicated days postinfection. Survival curves (A) and time course of parasitemia in nonpregnant *Tlr4*^−/−^ (B) and *Ifnar1*^−/−^ (C) females were compared with those of the wild type. Comparison of survival curves (A) using the log_10_ rank (Mantel-Cox) test showed nonsignificant differences (*P* > 0.05). For peripheral blood parasitemia of *Tlr4^−/−^* and *Ifnar1^−/−^* mice (B and C), no significant differences were detected in comparisons against the wild type by using a Kruskal-Wallis test with Dunn's correction for multiple comparisons.

We have shown previously that infection of pregnant C57BL/6 females with the P. berghei NK65 parasite strain induces pregnancy impairments with deleterious effects on the fetus, namely, markedly decreasing fetus viability and fetal weight (47). We chose this infection model to unravel the impact of the fetus-derived innate immunity receptors TLR4 and IFNAR1 on severe outcomes of MiP. We started by testing whether the progression of infection was altered in nonpregnant *Tlr4^−/−^* and *Ifnar1^−/−^* females and found no sensible differences in the courses of parasitemia up to 5 days postinfection or in the time of survival, with most females dying at between 22 and 32 days postinfection, regardless of the female genotype (Fig. 1). This was in contrast to the survival rates observed for *Rag^−/−^*, *Cd8a*^−/−^, and *Tcr*β^−/−^ mice (see Fig. S1 in the supplemental material) and allowed us to search for specific effects of TLR4 or IFNAR1 on MiP and to evaluate the role of fetal genotypes in the outcomes of MiP. To dissect the role of TLR4 or IFNAR1 expression in maternal versus fetal compartments, we compared the infection outcomes of heterogenic pregnancies (where mothers were deficient for the gene of interest [*Tlr4* or *Ifnar1*] but their fetuses were all heterozygous) against those of wild-type or gene-deficient isogenic pregnancies using the mating strategy described in Materials and Methods. Given that the P. berghei NK65 infection model induces abortions at the end of pregnancy, we used a previously validated methodology (47) and analyze fetuses and placentas at gestational day 18 (G18).

### Fetal TLR4 confers robust fetus survival but does not impact parasite burden.

**FIG 2:**
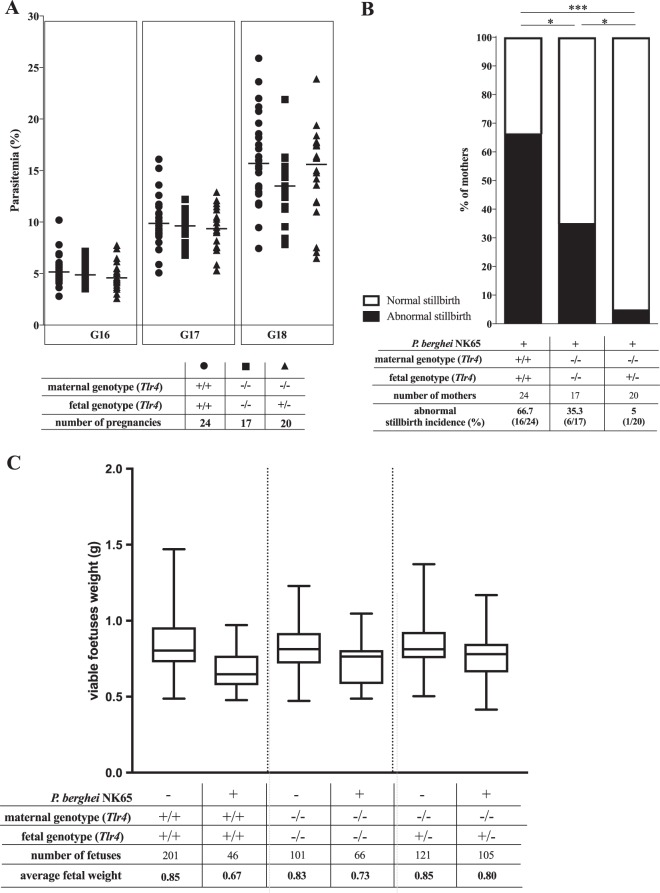
Effects of maternal versus fetal TLR4 on outcomes of malaria in pregnancy. Pregnant females of the indicated *Tlr4* maternal/fetal genotype combinations were infected i.v. at G13 with 10^6^ IEs. (A) Maternal parasitemia at G16 to G18 was determined by FACS analysis using DRAQ5-labeled samples. No significant differences were detected between genotype combinations by using a Kruskal-Wallis test with Dunn's correction for multiple comparisons. Data for each genotype group are presented as individual values and means. (B) Stillbirth incidence evaluated at G18. Data are presented as percentages of females showing an abnormal stillbirth rate (shadowed area) for each genotype combination. Abnormal stillbirth in individual females was determined when the stillbirth rate was above the maximum rate observed for the uninfected wild-type controls (stillbirth rates for individual females are depicted in Fig. S2 in the supplemental material). Differences in abnormal stillbirth incidences were analyzed by χ^2^ Fisher's exact test (*, *P* < 0.05; ***, *P* < 0.001). (C) Viable fetal weight is represented as a box and whisker plot, with whiskers extending to the minimum and maximum values obtained in each group. A linear mixed-effects model incorporating fetal genotype and maternal infection status was applied as described in Materials and Methods, showing that maternal infection status plays a significant role in the reduction of the weight of viable fetuses (*P* < 0.001), whereas fetal genotype does not (*P* = 0.65).

We monitored peripheral parasitemia after infection at G13 in wild-type females and in *Tlr4^−/−^* females carrying *Tlr4*^−/−^ or *Tlr4*^+/−^ fetuses and found that the *Tlr4* genotype in the maternal compartment did not affect the course of parasitemia during pregnancy, irrespective of the fetal *Tlr4* genotype (Fig. 2A). The impact of TLR4 on pregnancy outcomes was ascertained by comparing the proportions of abnormal stillbirths and individual fetus weights of the different maternal-fetal genotype combinations, 5 days after infection. As expected (47), the incidence of abnormal stillbirth induced by infection was very high among wild-type females (67%) (Fig. 2B). Surprisingly, the stillbirth rate was abnormal in only 5% of *Tlr4*^−/−^ mothers that carried *Tlr4*^+/−^ fetuses. The dramatically reduced stillbirth occurrence for this heterogenic combination was very close to that observed for noninfected controls (see Fig. S2 in the supplemental material). This was due mainly to a marked decrease in the number of females with a high stillbirth incidence. Nevertheless, this result is not fully attributable to the absence of maternal TLR4, as *Tlr4*^−/−^ isogenic pregnancies showed an abnormal stillbirth incidence of 35% (Fig. 2B). In fact, the very low stillbirth incidence for the heterogenic *Tlr4*^−/−^/*Tlr4*^+/−^ maternal-fetal combination implies that TLR4 acts in the fetal compartment to confer robust protection against increased stillbirth incidence in MiP. The reduction in fetal weight induced by infection was less pronounced in *Tlr4^−/−^* pregnant females than in wild-type pregnancies and was marginally recovered in *Tlr4^−/−^* pregnant females that carried fetuses expressing TLR4. Nevertheless, no significant difference was found in the weights of *Tlr4^−/−^* and *Tlr4^+/−^* fetuses when mothers lacked TLR4, suggesting that fetal TLR4 does not play a significant role in the weight reduction of viable fetuses in MiP (Fig. 2C). These findings indicate that the expression of TLR4 in fetal placental cells remarkably counteracts maternal TLR4 and contributes to the protection of fetus viability in the context of placental malaria.

### Fetal TLR4 contributes to the uptake of infected erythrocytes by trophoblast cells.

**FIG 3:**
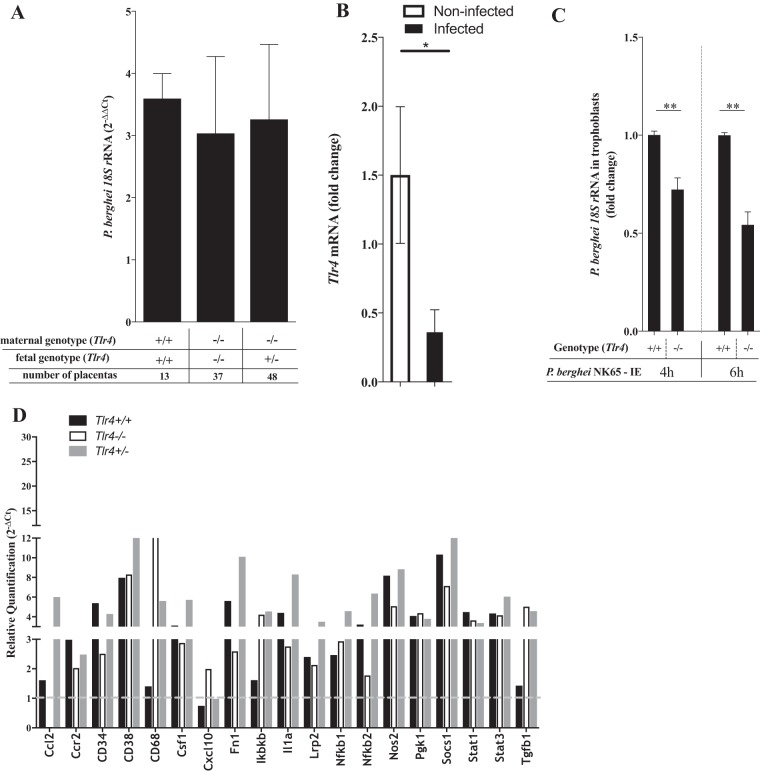
Fetal TLR4, placental infection, and trophoblast-infected erythrocyte interactions. (A) Pregnant females of the indicated maternal/fetal genotype combinations were infected, and placentas were collected at G18. The placental parasite burden was evaluated by quantification of P. berghei 18S rRNA levels in individual placentas by quantitative real-time PCR. Δ*C_T_* was calculated by subtracting the *C_T_* value of the target gene from that of the glyceraldehyde-3-phosphate dehydrogenase (GAPDH) gene. No statistical differences were detected by using a Kruskal-Wallis test with Dunn's correction for multiple comparisons. (B) Relative quantification of *Tlr4* mRNA expression levels in placentas from noninfected (*n* = 6) and infected (*n* = 6) wild-type females by quantitative real-time PCR (*, *P* < 0.05). (C) Trophoblast primary cultures from wild-type and *Tlr4^−/−^* placentas were prepared as described in Materials and Methods and exposed or not exposed to IEs (ratio, 1:1) during 4 or 6 h. P. berghei 18S rRNA levels in individual cultures were quantified by quantitative real-time PCR and are represented as fold increases relative to the values for wild-type cultures (**, *P* < 0.01). (D) Expression profiling of selected inflammation-related genes in infected placentas from wild-type (*Tlr4^+/+^*), TLR4 KO (*Tlr4^−/−^*), or heterogenic (*Tlr4^+/−^*) matings. RNA pools of 10 placentas of each genotype class were analyzed by using the TaqMan Array Mouse Immune panel and quantified by quantitative real-time PCR. Quantification results are relative to those of wild-type uninfected placentas and represent the means of data from two independent experiments.

We next asked whether the protection afforded by fetal TLR4 could be attributed to alterations in the course of infection in the placenta. We analyzed the placental parasite burden at G18 for the different maternal/fetal *Tlr4* genotype combinations. Interestingly, quantification of parasite RNA in the infected placentas indicated that neither maternal nor fetal TLR4 altered the placenta parasite burden (Fig. 3A), strongly suggesting that the fetal survival phenotype governed by TLR4 was not related to the amount of infected erythrocytes that accumulate in the placenta. Likewise, mRNA expression profiling of representative inflammatory genes in placentas from the different *Tlr4* genotype combinations showed no sound differences in the placental inflammatory response at G18. This suggests that the fetal protection mechanism conferred by fetal TLR4 does not alter the placental inflammatory milieu substantially (Fig. 3D). Nevertheless, we noted that the expression of the SOCS1 gene in infected placentas correlated with fetus protection in heterogenic pregnancies of *Tlr4^−/−^* animals carrying *Tlr4^+/−^* fetuses. Given that SOCS1 is involved in the negative regulation of cell activation by TLR4 signaling (48, 49) and TLR4-dependent phagocytosis (50, 51), this finding suggests that malaria infection may induce a state of TLR4 tolerization (52) in fetal placental cells. In agreement, we found that TLR4 gene expression was downregulated in infected placentas from wild-type mice (Fig. 3B). To ascertain the role of fetal TLR4 in the response to infection, we used primary cultures of trophoblasts isolated from noninfected placentas with wild-type or *Tlr4^−/−^* genotypes. We found that infected erythrocytes are taken up by trophoblast cells, as measured by P. berghei RNA levels in trophoblasts after exposure to infected erythrocytes for 4 or 6 h (Fig. 3C). Additionally, we show that the expression of TLR4 in trophoblasts significantly contributes to the uptake of infected erythrocytes, indicating that fetal TLR4 takes an active part in the trophoblast response to placental malaria infection.

### Maternal IFNAR1 and fetal IFNAR1 have opposing actions in the accumulation of intraplacental parasites.

**FIG 4:**
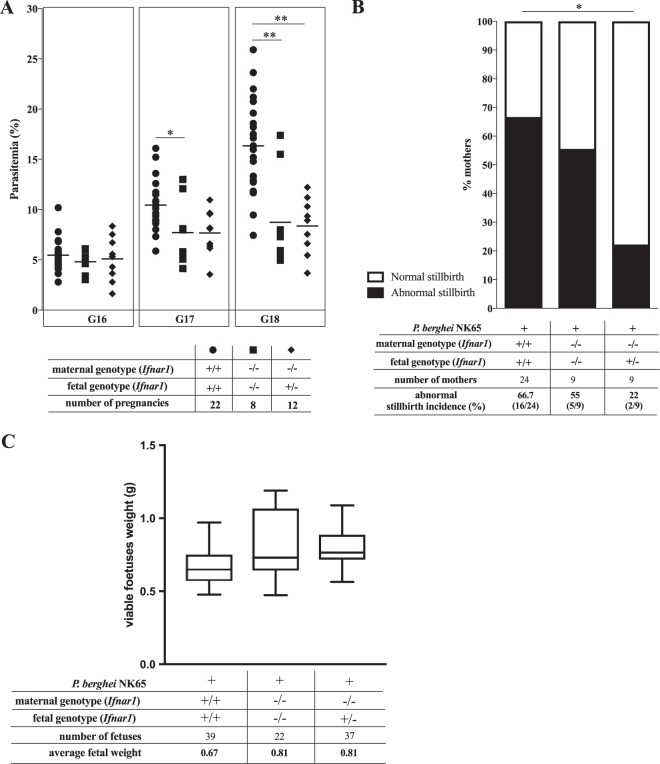
Maternal versus fetal IFNAR1 effects on outcomes of malaria in pregnancy. Maternal P. berghei NK65 parasitemia at G16 to G18 (A) and abnormal stillbirth incidences (B) and weights of viable fetuses (C) at G18 were analyzed for the indicated maternal-fetal *Ifnar1* genotype combinations as described in the legend of Fig. 2. The presentation of data is analogous to that for Fig. 2 (stillbirth rates for individual females are depicted in Fig. S2 in the supplemental material). Detection of statistical differences between genotype combinations was done by using a Kruskal-Wallis test with Dunn's correction for multiple comparisons (A), χ^2^ Fisher's exact test (B), or a linear mixed-effects model approach incorporating either fetal or maternal genotype as a fixed effect alongside a random effect for each mother (C). Using type III ANOVAs with Satterthwaite approximation for degrees of freedom, we find that fetuses in infected mothers who are *Ifnar^−/−^*, irrespective of the fetal genotype, show a trend toward higher weight than those of their counterparts carried by infected *Ifnar^+/+^* mothers (*P* = 0.087) (C). *, *P* < 0.05; **, *P* < 0.01.

The course of infection in pregnant *Ifnar1^−/−^* females showed lower levels of peripheral parasitemia than those for the wild type, irrespective of the fetal *Ifnar1* genotype (Fig. 4A), indicating that IFNAR1-mediated responses in the maternal compartment contribute to the development of hyperparasitemia during pregnancy. These results are in line with data from previous studies claiming that in hyperparasitemia models, the progression of infection is attenuated in IFNAR1 knockout (KO) mice (37). Reportedly, *Ifnar1*^−/−^ females have fertility success and litter sizes similar to those of wild-type mice, indicating that fetus viability is not impaired by a lack of IFNAR1 expression (53). We observed that fetus survival at G18 in infected *Ifnar1*^−/−^ females was not significantly altered compared to that in wild-type females, and the *Ifnar1*^−/−^/*Ifnar1*^+/−^ maternal-fetal combination offered only modest protection against fetus death, but owing to the constraints in group sizes, we cannot exclude that fetal IFNAR1 has a beneficial effect on fetus survival (Fig. 4B). Likewise, analysis of fetal weight suggested that the absence of maternal IFNAR1 showed only a trend (*P* = 0.087) toward opposing the weight loss of viable fetuses in infected females (Fig. 4C). Together, these results suggest that the maternal IFNAR1 action in MiP pathogenesis favors increased peripheral parasitemia and possibly contributes to fetal weight loss.

**FIG 5:**
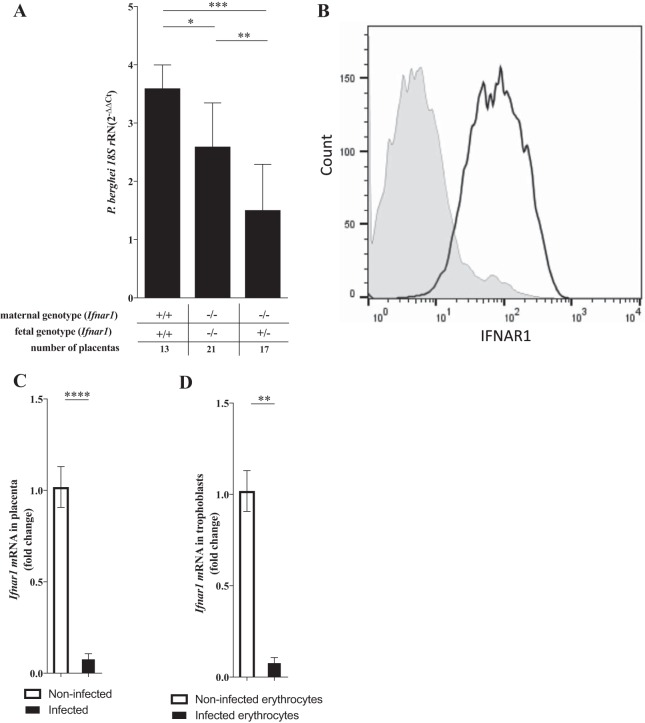
Fetal IFNAR1 in placental infections and in trophoblasts. (A) The placental parasite burden in individual placentas was estimated at G18 for the indicated maternal/fetal genotype combinations by quantifying P. berghei 18S rRNA levels as described in the legend of Fig. 2. (B) IFNAR1 surface expression in freshly isolated trophoblasts from noninfected placentas was detected by FACS analysis (empty histogram, staining with anti-IFNAR1 antibody; filled histogram, isotype control). (C and D) Relative quantification of *Ifnar1* mRNA gene expression levels in infected (*n* = 6) versus noninfected (*n* = 6) placentas (C) and in primary trophoblast cultures from wild-type placentas exposed or not exposed to P. berghei NK65-infected erythrocytes during 4 h (D) by quantitative real-time PCR. *, *P* < 0.05; **, *P* < 0.01; ***, *P* < 0.001; ****, *P* < 0.0001.

Although the placental parasite load was also significantly reduced in *Ifnar1^−/−^* mothers, this effect was most prominent when *Ifnar1^−/−^* mothers carried *Ifnar1*^+/−^ fetuses (Fig. 5A). These observations indicated that maternal and fetal IFNAR1 have opposing actions in the accumulation of intraplacental parasites in MiP. We analyzed freshly isolated placental cells from noninfected placentas by fluorescence-activated cell sorter (FACS) analysis and found that IFNAR1 is widely expressed in fetal trophoblasts (Fig. 5B). Furthermore, the expression levels of the *Ifnar1* gene were dramatically reduced in infected placentas and in trophoblast primary cultures exposed to infected erythrocytes (Fig. 5C and D). These results suggest that the interferon-IFNAR1 signaling axis is modulated during infection, with an impact on the accumulation of infected erythrocytes in the placenta. In conclusion, this work uncovers fetal innate immunity receptors that play a role in controlling the outcomes of placental malaria through reducing the placental parasite burden or by improving fetus survival.

## DISCUSSION

We have investigated the role of TLR4 and IFNAR1 in malaria during pregnancy, with a focus on dissecting the contributions of maternal and fetal compartments to pregnancy outcomes. To this end, we compared wild-type and null-mutation pregnancies with heterogenic pregnancies in which the mother and fetuses carry different genotypes with regard to the gene of interest. This allowed us to analyze the isolated effects of TLR4 and IFNAR1 in the fetal compartment in the absence of the same gene in the maternal compartment. We found that fetal *Tlr4* and *Ifnar1* genes show protective effects during the course of infection, opposing the pathogenic action of the maternal counterparts.

Maternal TLR4 did not impact the development of MiP when disease severity was evaluated by the levels of peripheral parasitemia and placental parasite burden. Nevertheless, infection in *Tlr4^−/−^* pregnant females was less deleterious to the fetuses, partially restoring the stillbirth incidence. This clearly indicates that maternal TLR4 is a pathogenesis factor in MiP that contributes to poor pregnancy outcomes but that it does not operate by controlling the parasite burden. This observation is in line with data from previous reports showing that during bacterial infections, the maternal immune system severely impairs pregnancy outcomes in a TLR4-dependent manner (22–24), with no impact on the placental bacterial load (23).

Unexpectedly, we uncovered that the expression of TLR4 in the fetal compartment led to a reduction in the incidence of malaria-induced fetus loss *in utero*. Moreover, this protective effect was not paralleled by a reduction in the level of maternal parasitemia or in the placental parasite burden, suggesting that protection of fetus viability by fetal TLR4 did not rely on antiparasite responses. It should be noted that the identification that fetal TLR4 protects fetus viability relies on observations made for hemizygous *Tlr4* expression (*Tlr4^+/−^* placentas), where TLR4 expression is conferred only by the paternal allele. Therefore, it is conceivable that the homozygous expression of fetal *Tlr4* in the absence of maternal TLR4 would have stronger fetus protection phenotypes. Interestingly, this protection effect was detected only when pregnant females lacked *Tlr4*, suggesting that in wild-type pregnancies, the pathogenic effects of maternal TLR4 override fetal TLR4 protection. Our data further suggest that fetal TLR4 participates in the uptake of infected erythrocytes, leading to alterations in gene expression that are compatible with trophoblast tolerization. We speculate that this tolerization state governs the response of fetal placental cells to infection, preserving the otherwise impaired placenta function but with little effect on parasite clearance. While it has been shown that nutrient transport is impaired in MiP (54, 55), there is still little evidence on the mechanisms that alter placental physiology during infection (56). Nevertheless, it has been shown that the fetus reacts to placental malaria infection with a strong proangiogenic response that is counteracted by maternal innate immune components. This fetal response provides an example where the restoration of fetal weight is not accompanied by a reduction of parasitemia levels (57). Likewise, we hypothesize that fetal TLR4 expressed at the maternal-fetal barrier acts as a sensor that initiates fetal protection responses leading to a reinforcement of placental function, with no influence on placental burden. It remains to be determined whether this TLR4 response is operating in other placental infections and how it conveys fetal protection.

In contrast to TLR4, the action of maternal IFNAR1 was correlated with high peripheral and placental parasite burdens and may contribute to reduced fetal weight. This is in line with previous observations that associate type I interferon signaling with an impaired control of parasite expansion and restricted antibody responses to malaria parasites (37, 58). Thus, it has been proposed that the ability to mount early parasite-specific B cell responses in lymphatic follicles is reduced because IFNAR1 signaling in DCs limits the activation and accumulation of CD4 T helper cells(59, 60) or because IFNAR1 signaling in CD4 T cells promotes Tr1 expansion (37, 61). On the other hand, when infection is established, the production of type I interferon by plasmacytoid DCs leads to enhanced IFNAR signaling in conventional DCs, which in turn drives protective responses and improved infection outcomes (31). We also found that fetal IFNAR1 counteracted placental parasite accumulation, suggesting that type I interferon signaling in placental cells leads to an improvement in antiparasite responses. In addition, our results also show that IFNAR expression is highly reduced in infected placentas and in trophoblasts exposed to infected erythrocytes. These results raise the possibility that interferon signaling in trophoblasts is counteracted during the course of infection, leading to the downregulation of IFNAR1 gene expression. Thus, it is plausible that the reduction of the parasite burden attributable to fetal IFNAR1 is not large enough to significantly prevent placental insufficiency and the subsequent detrimental effects on the fetus.

Our findings on the involvement of maternal TLR4 and IFNAR1 in MiP highlight that the expression of these innate immunity mediators in the maternal compartment has a deleterious role in outcomes of MiP. Accordingly, a large body of evidence indicates that inflammatory cells and mediators that take part in maternal responses during MiP exacerbate placenta pathology (12, 62, 63) or are detrimental to the fetus by impairing placenta function at the levels of maternal blood flow (57, 64) and nutrient uptake (54).

Evidence that *in vitro* trophoblast responses may play a role in enhancing the inflammatory milieu and placental tissue damage during MiP was reported previously (65–69). Additionally, it has been shown that trophoblasts express an array of innate immunity receptors, including TLRs and NOD-like receptors (70–74), and mediate interferon production (75), suggesting that these cells, located at the maternal-fetal interface, are equipped to sense and respond to innate immunity triggers. Our results showing that TLR4 and IFNAR1 in the placenta have fetus-protective roles reveal an unexpected outcome of the action of innate receptors that opposes the effects of maternal receptor counterparts and thereby expose a maternal-fetal conflict in the response to infection. Our observations imply that the molecular wiring and cellular responses to innate immunity stimuli are substantially different in trophoblasts compared to the maternal innate immune system and compose part of a fetal protective mechanism that promotes placental function. Furthermore, our results revealed that the protection of fetus viability conferred by fetal innate immunity factors such as TLR4 is not connected to a reduction of the parasite burden. This finding suggests that the severe consequences of MiP could be lessened if fetal protective mechanisms are pharmacologically enhanced, in parallel with antiparasite therapeutic strategies.

## MATERIALS AND METHODS

### Mice and pregnancy monitoring.

C57BL/6 (WT), C57BL/6.Tlr4^−/−^ (*Tlr4^−/−^*), and C57BL/6.Ifnar1^−/−^ (*Ifnar1^−/−^*) mice aged 8 to 12 weeks were obtained from the animal facility at the Instituto Gulbenkian de Ciência. Mice were bred and maintained under specific-pathogen-free conditions. Isogenic matings were established as follows. WT, *Tlr4^−/−^*, or *Ifnar1^−/−^* females were transferred to a cage with one isogenic male (two females to one male). For heterogenic mating, null mutant females were transferred to a cage with wild-type males. Females were removed after 48 h, and this time point was considered G1. Pregnancy was determined by weighing the females every other day. Successful gestation was confirmed at G13 when females had an increase in body weight of 3 to 4 g. Abrupt weight loss after G13 was an indicator of unsuccessful pregnancy. Procedures using live animals were approved by the Instituto Gulbenkian de Ciência ethical committee and by the national animal welfare authority (DGAV) and were carried out in accordance with national (portaria 1005/92) and European (European Directive 56/609/CE) regulations.

### Parasites and infection.

In this study, we used a parasite line originally derived from P. berghei isolate NK65 at New York University, kindly provided by Maria Mota (Instituto de Medicina Molecular, Lisbon, Portugal). Frozen IE stocks were expanded in C57BL/6 mice prior to infection. Infections in Fig. 1 were performed by intraperitoneal (i.p.) injection of 10^6^ IEs. For other experiments, pregnant mice and the respective nonpregnant female controls were intravenously (i.v.) injected with 10^6^ infected erythrocytes. Parasitemia was measured by flow cytometry (76) to detect infected erythrocytes stained with DRAQ5 (Biostatus Limited). The method used for the labeling of infected red blood cells with DRAQ5 is an adaptation of the manufacturer's protocol for cell cycle analysis by flow cytometry. Briefly, a drop of blood was collected, by tail pinching of infected mice, into 400 μl of FACS buffer (1× phosphate-buffered saline [PBS], 2% fetal bovine serum [FBS], 0.02% sodium azide). DRAQ5 was added directly to the collected samples at a final concentration of 1 μM. Samples were mixed by vortexing to allow the appropriate incorporation of DRAQ5 into parasite DNA and were immediately analyzed. Parasitemia was expressed as the percentage of stained cells within the erythrocyte morphological gate. The time of infection and amounts of parasites were in accordance with those used in a previously characterized model of MiP in C57BL/6 females infected with P. berghei NK65 parasites (47).

### Pregnancy outcome and fetus survival.

Infected pregnant mice were killed by CO_2_ narcosis and subjected to caesarian section at G18, and fetus weight and viability were evaluated. Fetuses were extracted from their amniotic sac, and viability was immediately evaluated by reaction to touching with pliers. The lack of prompt movement indicated that the fetus had recently died. Resorptions were identified as small implants with no discernible fetus and placenta. To capture different causes of fetal death *in utero*, nonviable fetuses (dead fetuses plus reabsorptions) and fetuses that had been expelled before the gestational day of analysis were recorded as stillbirths. The stillbirth rate for individual females was calculated as the number of stillbirths/total number of fetuses. The weight of viable fetuses was recorded.

### Placenta preparations, RNA isolation, and gene expression analysis.

Placentas from infected and noninfected females sacrificed on the same gestational day (G18) were collected in lysis buffer (RNeasy minikit; Qiagen)–1% β-mercaptoethanol for RNA extraction. Total RNA from individual placentas was obtained by using an RNeasy minikit (Qiagen), according to the manufacturer's instructions. Equal amounts of each RNA sample were converted to cDNA (Transcriptor First Strand cDNA synthesis kit; Roche). The numbers of P. berghei parasites were quantified by using 18S RNA TaqMan assays with the following specific primers and probe: forward primer 5′-CCG ATA ACG AAC GAG ATC TTA ACC T-3′, reverse primer 5′-CGT CAA AAC CAA TCT CCC AAT AAA GG-3′, and probe 5′-ACT CGC CGC TAA TTA G-3′ (6-carboxyfluorescein [FAM]/MGB). The endogenous control gene *Gapdh* (mouse GAPD Endogenous Control, catalog no. 4352339E; ABI) was used in multiplex PCR assays with the target gene (*Tlr4* or *Ifnar1*). PCRs were performed with an ABI Prism 7900HT system. Δ*C_T_* was calculated by subtracting the cycle threshold (*C_T_*) of the target gene from the *Gapdh C_T_*. Results are presented as fold changes (2^−ΔΔC_T_^) relative to the values for the controls. Outliers were systematically excluded by using the ROUT method in GraphPad Prism software. Parasite burden in placentas is presented as P. berghei 18S RNA levels normalized to *Gapdh* levels and plotted as 2^−ΔΔC_T_^. For the gene expression profiling experiments with the TaqMan Array Mouse Immune panel, the arrays were loaded with pooled placental RNA (*n* = 10) from noninfected or infected females of one of the different maternal/fetal *Tlr4* genotype combinations. The results for each gene are reported as the level for each genotype combination relative to that for the respective noninfected control. The results presented represent the means of relative quantifications from two independent experiments.

### Isolation and characterization of primary trophoblasts.

Pregnant C57BL/6J and C57BL/6J.Tlr4^−/−^ females were sacrificed by carbon dioxide narcosis at G18, and the placentas were retrieved by caesarian section. The G18 placentas from each individual mother were pooled, and cells were dispersed in digestion medium (20 mM HEPES, 0.35 g/liter sodium bicarbonate, 1 mg/ml collagenase type 1 [catalog no. C9891; Sigma], 1 20 μg/ml DNase [catalog no. 11284932001; Roche]) and incubated at 37°C for 1 h. The digestion mixture was then passed through a 70-μm cell strainer to remove large clumps and centrifuged at 500 relative centrifugal force (RCF) for 5 min to pellet the cells. The cells were resuspended in 4 ml of 25% Percoll (catalog no. 17-0891-01; GE Healthcare) in RPMI medium and layered onto 4 ml of 40% Percoll, and a final 2-ml layer of PBS was placed on top. After centrifugation at 800 RCF for 20 min with no break, the interface with trophoblasts was collected, washed, and resuspended in 1 to 2 ml of RPMI complete medium. The cells were counted and either plated onto 96-well plates (10^6^ cells per well) and incubated at 37°C for 7 to 10 days or used for flow cytometry analysis.

### Trophoblast FACS analysis.

The purity of the trophoblast cultures was assessed by the expression of the trophoblast marker cytokeratin-7 (KRT7), detected by FACS analysis. A sample of cells was taken before plating, and a sample of cultured cells was retrieved from the plate before further use. The cells were washed with FACS buffer and processed for intracellular staining with anti-KRT7 antibodies (RCK105, catalog no. sc-23876; Santa Cruz Biotechnology Inc.). Typically, preparations of cultured cells were >85% KRT7^+^. For IFNAR1 surface staining in placental cells, placentas were obtained from crossing C57BL/6J females with B6.Actin-GFP males, where fetal cells are green fluorescent protein positive (GFP^+^). Single-cell suspensions were prepared in the presence of phycoerythrin (PE)-labeled anti-IFNAR-1 monoclonal antibody (clone MAR1-5A3; BioLegend). FACS acquisition was carried out on a BD LSR Fortessa X-20 instrument (Becton, Dickinson and Company), and FlowJo software (Tree Star) was used to analyze the mean fluorescence intensity (MFI) of IFNAR within GFP^+^ cells in the trophoblast morphological gate.

### Statistical analysis.

Survival curves were compared by using the log rank (Mantel-Cox) test. A Kruskal-Wallis nonparametric test with Dunn's multiple-comparison test was used for comparisons of peripheral parasitemia and placental parasite load. Comparisons of abnormal stillbirth incidences were analyzed with χ^2^ Fisher's exact test. Fetal weight was assessed with a linear mixed-model approach, which incorporated fetal genotype and maternal infection status as fixed effects alongside a random effect accounting for variation within each litter; analysis of variance (ANOVA) with Satterthwaite approximation for degrees of freedom was performed; and Akaike and Bayesian information criteria were also obtained and compared. This analysis was performed by using the lme4 and lmerTest packages available for R (77–79). Data were considered significant when the *P* value was <0.05. Power calculations from the abnormal stillbirth incidence analysis indicate that the powers of this study to detect a 50% decrease in abnormal stillbirth incidence when comparing maternal/fetal genotype combinations were 89% for WT versus *Tlr4* KO, 91% for WT versus *Trl4* KO/WT, 86% for *Tlr4* KO versus *Trl4* KO/WT, 82% for WT versus *Ifnar1* KO, 82% for WT versus *Ifnar1* KO/WT, and 60% for *Ifnar1* KO versus *Ifnar1* KO/WT.

## Supplementary Material

Supplemental material
