# Supplementary material for: Fetal and Maternal Innate Immunity Receptors Have Opposing Effects on the Severity of Experimental Malaria in Pregnancy: Beneficial Roles for Fetus-Derived Toll-Like Receptor 4 and Type I Interferon Receptor 1
Source: Infect Immun. 2018 Apr 23;86(5):e00708-17. doi: 10.1128/IAI.00708-17 (PMC5913849; doi:10.1128/IAI.00708-17)
Supplement: Supplemental material [file supp_86_5_e00708-17__index.html]

Supplemental material 

# Fetal and Maternal Innate Immunity Receptors Have Opposing Effects on the Severity of Experimental Malaria in Pregnancy: Beneficial Roles for Fetus-Derived Toll-Like Receptor 4 and Type I Interferon Receptor 1

## Supplemental material

- Supplemental file 1 -

  Fig. S1. Susceptibility to infection of Rag2−/−, Cd8a−/−, and Tcrβ−/− nonpregnant females. Fig. S2. Stillbirth rates in individual females of different *Tlr4* or *Ifnar1* genotype combinations.

  PDF, 3.1M
