# Supplementary material for: Fetal and Maternal Innate Immunity Receptors Have Opposing Effects on the Severity of Experimental Malaria in Pregnancy: Beneficial Roles for Fetus-Derived Toll-Like Receptor 4 and Type I Interferon Receptor 1
Source: Infect Immun. 2018 Apr 23;86(5):e00708-17. doi: 10.1128/IAI.00708-17 (PMC5913849; doi:10.1128/IAI.00708-17)
Supplement: Supplemental material [file IAI.00708-17_zii999092383s1.pdf]

Foetal and maternal innate immunity receptors have opposing effects in severity of experimental malaria in pregnancy: Beneficial roles for foetal-derived TLR4 and IFNAR1.

Lurdes Rodrigues-Duarte, Yash Pandya, Rita Neres and Carlos Penha-Gonçalves

Supplemental Material

Supplemental Figure 1

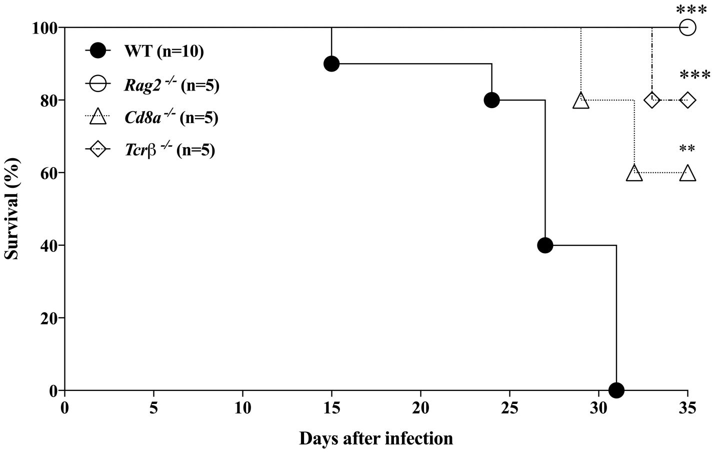

Supplementary Figure 1. Susceptibility to infection of Rag2-/-, Cd8a-/- and Tcrβ-/- non-pregnant females.

(A) Survival curves of *P. berghei* NK65 infected wild-type (B6), *Rag2*$^{-/-}$, *Cd8a*$^{-/-}$ and *Tcrβ*$^{-/-}$ non-pregnant females. Animals were infected i.p. with 10^6 IE. Parasitaemia of DRAQ-5 labeled samples was analyzes by FACS. Survival curves were compared using the Log-Rank (Mantel-Cox) test. Significance levels: **p<0.01), (***p<0.001).

Supplemental Figure 2

A

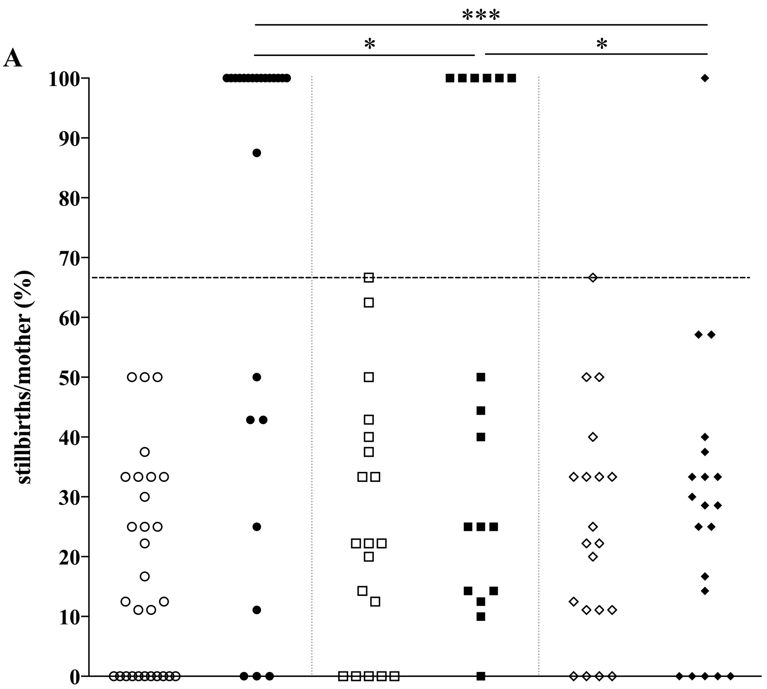

P. berghei NK65	-	+	-	+	-	+
**maternal genotype (*Tlr4*)**	+/+	+/+	-/-	-/-	-/-	-/-
**foetal genotype (*Tlr4*)**	+/+	+/+	-/-	-/-	+/-	+/-
**number of mothers**	29	24	19	17	20	20
**abnormal stillbirth incidence %**	n.a.	66.7 (16/24)	n.a.	35.3 (6/17)	n.a.	5 (1/20)

Horizontal dashed lines indicate the maximum stillbirth rate observed in each of the non infected control groups. Stillbirths/mother in infected mothers that fell below this value were deemed normal and those above abnormal.

B

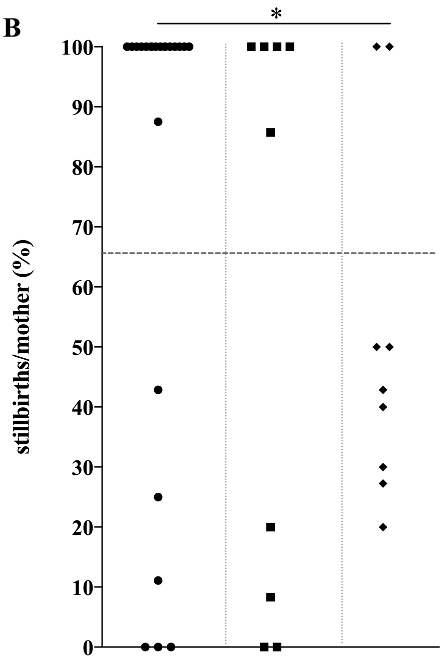

P. berghei NK65	+	+	+
maternal genotype (*Ifnar1*)	+/+	-/-	-/-
foetal genotype (*Ifnar1*)	+/+	-/-	+/-
number of mothers	23	9	9
**abnormal stillbirth incidence %**	**73.9 (16/23)**	**50.0 (5/9)**	**22.2 (2/9)**

Supplementary Figure 2. Stillbirth rate in individual females of different *Tlr4 or Ifnar1 genotype combinations.*

Stillbirth incidence was accessed at G18 for the indicated *Tlr4* **(A)** and *Ifnar1* **(B)** maternal/foetal genotype combinations. Stillbirth percent in individual females was calculated as the number of stillbirths /number of total foetuses X 100. Cut-off for abnormal stillbirth incidence was established considering the maximum number of stillbirths in non-infected pregnant mice (dashed line).
